# Health-related quality of life among Syrian refugees resettled in Sweden

**DOI:** 10.1007/s11136-019-02323-5

**Published:** 2019-10-15

**Authors:** Maria Gottvall, Sara Sjölund, Charlotta Arwidson, Fredrik Saboonchi

**Affiliations:** 1 Department of Health Sciences, The Swedish Red Cross University College, PO Box 1059, 141 21 Huddinge, Sweden; 2 Department of Women’s and Children’s Health, Uppsala University, Uppsala, Sweden; 3 Division of Insurance Medicine, Department of Clinical Neuroscience, Karolinska Institutet, Stockholm, Sweden

**Keywords:** Quality of life, Refugees, Social support, Syria, Resettlement

## Abstract

**Purpose:**

The main purpose of this study was to assess health-related quality of life (HRQoL) among Syrian refugees resettled in Sweden. Further, we wanted to investigate whether sex, age, education, area of residence, cohabitation and social support were associated with HRQoL in this population.

**Methods:**

This is a cross-sectional study including 1215 Syrian refugees from a randomly selected sample frame resettled in Sweden between the years 2011 and 2013. HRQoL was measured by the EQ-5D-5L descriptive system, and EQ-5D-5L index values were calculated. Associations between sex, age, education, area of residence, cohabitation, social support and EQ-5D-5L were investigated using multiple linear regression analysis.

**Results:**

Depression/anxiety was the most commonly (61.9%) reported EQ-5D-5L problem among the group of Syrian refugees. The mean EQ-5D-5L index value was found to be 0.754. Male sex, younger age, cohabitation and social support were found associated with a higher EQ-5D-5L index score.

**Conclusions:**

Our results concerning long-lasting health problems among the study population indicate that there is a profound need for policies and interventions promoting refugees’ health. Our results also show that social support, a modifiable factor, is relevant to refugees’ overall health, pointing to the importance of public health interventions and policies targeting the facilitation, mobilization and enhancing of refugees’ social support.

## Introduction

The world is now facing the highest number of forcibly displaced people ever recorded—65 million people. In 2015, a year when a high number of migrants arrived in Europe, Sweden received 163,000 applications for asylum. The greatest share of these applicants originated from Syria [1].

Potentially traumatic experiences, e.g., war at close quarters, are common among refugees coming from war-torn regions [2, 3], and many refugees may suffer from mental ill health due to these experiences [2, 4, 5]. Prevalence of mental ill health such as anxiety, depression and PTSD seems to be high among war refugees [2, 4–7]. Specifically, high prevalence of mental ill health has been found in a large random sample of Syrian refugees resettled in Sweden [2]. Given that health, as defined by WHO [8] is an integrated concept including mental aspects as well as physical and social well-being, a too narrow focus on mental health may, however, conceal a broader understanding of refugee health and how health impacts the lives of refugees in a broader sense. Health-related quality of life (HRQoL) is a measure closely related to the WHO’s definition of health as it takes into account several dimensions of health and thus warrants to be a target of examination in this context.

Refugees’ health is a highly gendered area. Women are exposed to particular health risks in the migration process such as sexual violence and exploitation. Previous research has shown that refugee women report both more physical and more mental problems than refugee men [4, 9]. Research focusing on refugees’ health therefore needs to include specific gender analyses [10]. Besides gender, age is important for health among refugees. Older refugees report more mental ill health [2, 9], as well as worse physical functioning [11] than younger refugees.

Social support and networks are, in general, considered important protective factors relating to both mental and physical health [12, 13]. Social support has been described as the social resources that persons perceive to be available or that are actually provided to them by nonprofessionals in the context of both formal support groups and informal helping relationships [14]. Refugees’ social networks are often upheaved due to migration, and rebuilding social networks and ties may prove to be challenging.

There are few previous studies published regarding the HRQoL of refugees resettled in high-income countries. Results from a study in Finland among older Somali refugees showed that they had a lower HRQoL than the matched Finnish host population. Somali refugees reported more on the dimension of anxiety/depression, whereas the Finnish control group reported more on pain/discomfort [15]. The HRQoL in elderly has also been investigated among Iranian refugees resettled in Sweden indicating that Iranian refugees in Sweden reported higher HRQoL than Iranians living in Iran, but lower than native Swedes living in Sweden [16].

Today, there is a lack of robust knowledge about the general health status of refugees. In particular, knowledge about the health status of Syrian refugees resettled in a high-income country is scarce. In order to provide effective and useful health-related interventions adapted for men, women or for different age groups, it is important to assess the health status in this refugee population, how it is distributed within the group and other factors it could be influenced by. Therefore, the aim of this study was to assess the HRQoL among Syrian refugees resettled in Sweden, in total and stratified by sex and age group, and also to investigate whether there is an association between HRQoL and sex, age, educational level, cohabitation, place of residence, and social support in this group.

## Methods

### Study design and participants

This is a cross-sectional study based on data from a randomized sample of Syrian refugees resettled in Sweden. Eligible for the study were all men and women born between 1952 and 1998 in Syria, who were granted permanent residency in Sweden on grounds of asylum between 2011 and 2013. A sampling frame of 9662 individuals was identified through the Total Population Register (TPR) held by Statistics Sweden, which carries information on vital status on all individuals with permanent residency in Sweden. Information on date for residence permit and reason for residence was retrieved from the database STATIV, also held by Statistics Sweden.

A simple random sample of 4000 women and men, aged 18–64 years, was drawn by Statistics Sweden from the sampling frame of the 9662 individuals identified through TPR. A postal questionnaire in Arabic with the aim to collect information about mental health and factors related to mental health was sent by Statistics Sweden to the random sample, in February 2016. Two postal reminders were sent in March 2016. As a third reminder, a phone call was made for those in the sample who had not responded to the questionnaire after the second postal reminder. The phone call was made by an Arabic speaking person from the Red Cross University College. Data collection ended in April 2016.

The study population of this study constitutes the 1215 individuals who responded to the questionnaire. The procedure regarding language and usability of the questionnaire is described in more detail in Tinghög et al. [2].

### Variables

#### Health-related quality of life

The EuroQol-5D-5L (EQ-5D-5L) was used as a measure of (HRQoL). This instrument consists of a descriptive system including the following five dimensions: mobility, self‐care, usual activities, pain/discomfort and anxiety/depression, and each dimension has five levels: no problems, slight problems, moderate problems, severe problems and extreme problems. The respondent’s answer on each of the five dimensions results in a one-digit number indicating the level selected for that dimension. The digits for the five dimensions are combined in a five-digit number describing the respondent’s health state. The health states may be converted into a single index value between zero and one, where zero is equal to death and one is equal to a state of perfect health, through an “index-calculator” [17]. The health profiles in this study were converted to the index value from the EQ-5D crosswalk tariff from the UK. There are no index tariffs for the Swedish population, and the UK index tariff is frequently used in population-based studies in Sweden [18, 19]. It is a value set which is based on hypothetical values, in contrast to experience-based value sets [19].

The instrument also consists of the EQ visual analogue scale (EQ VAS). However, due to technical difficulties involved in scanning the visual analogue scale (VAS) properly, we were not able to acquire data from the VAS section and consequently VAS was not included in the analysis. The EQ-5D-5L has been shown reliable and valid in Arabic [20].

#### Socio-demographic variables

Socio-demographic variables used in this study were age, sex, civil status, educational level and place of residence. Information on socio-demographic variables was retrieved from the TPR database, except for civil status which was retrieved from the postal questionnaire.

In our analysis, age was categorized into groups: 18‒29 years, 30‒39 years, 40‒49 years and 50‒64 years. Civil status was grouped into (1) cohabitant, which included unmarried (living with a partner), and married (currently living with husband/wife) and (2) not cohabitant which included unmarried (not living with a partner), married (not currently living with husband/wife), divorced and widow/widower.

Educational level was categorized into number of years of schooling: 0‒9 years, 9‒12 years and > 12 years. There were 33 individuals (16 women and 17 men) with missing data on educational level. They were included in the category 0‒9 years of education. For place of residence, definitions of regions by the Swedish Association of Local Authorities and Regions (SALAR) were used: (1) Big city: municipality with > 200,000 inhabitants and municipality at commuting distance to a big city, (2) Town: municipality with > 50,000 habitants, with > 40,000 habitants in the largest town or municipality at commuting distance to a town) and (3) Smaller villages: < 40,000 inhabitants.

#### Social support

To measure Social support, ENRICHD Social Support Inventory (ESSI) was used. ESSI is a short, seven-item, self-administered instrument that provides a single score of social support covering three different types of support—structural, instrumental and emotional [25]. The items assess whether there is someone available to the participant who will listen, give advice, show love/affection, help with daily chores, provide emotional support and can be confided in. On these six items, the response is rated on a five-point Likert scale that ranges from *None of the time* to *All of the time.* The seventh item *Are you currently married or living with a partner?* is answered with *Yes* or *No*. Low social support is defined according to ESSI criteria 2—a score of three or less on two or more items and a total score of ≤ 18 (not including items *help with daily chores* and *civil status*) [21]. ESSI has been validated for use among Syrian refugees [22].

### Statistical analysis

The statistical analysis was performed using the IBM SPSS statistics 25. Descriptive statistics regarding both outcome and covariates generated prevalence proportions. Prevalence proportions for the five dimensions of EQ-5D-5L both in total and stratified by sex were calculated. Mean, median, 25th, 75th percentiles and standard deviation were calculated for the EQ-5D-5L index values, in total and stratified by sex and age. To test for differences in reported problems in the five dimensions between women and men and between different age groups logistic regression analysis was used. For this analysis, the scale was dichotomized into the categories *No problems* (Level 1) and *Slight problems* to *Unable to perform/extreme problems* (Level 2‒5). For assessing the association between EQ-5D-5L and the variables sex, age, educational level, cohabitation, place of residence and social support, multiple linear regression was used. EQ-5D-5L index values were treated as continuous outcomes in three different models. Explained variances for each model, R^2^, was computed to provide a measure of the contribution of each added predictor in the regression models.

## Results

A majority (56%) of the respondents were between 18 and 39 years, and more than a third of the respondents (37%) were women (Table 1). More than half (60%) had attended school for more than nine years, and the vast majority (94%) had arrived in Sweden 3‒4 years before the data collection. Almost a third of the population (28%) were living in or at a commuting distance from one of the three big cities in Sweden (Stockholm, Gothenburg and Malmoe). Approximately two-thirds of the respondents (59%) reported low social support.

**Table 1:** Descriptive statistics of the respondents (*n* = 1215) supplemented with non-response analysis

	*n* (%)	Women/men *n*	Non-respondents *n* (%)	Respondents vs. non-respondents χ^2^ (*P* values)
Sex				0.4 (0.52)
Women	452 (37.2)		1008 (36.2)	
Men	763 (62.8)		1777 (63.8)	
Age groups				68.7 (< 0.01)
18–29	283 (23.3)	122/161	947 (34.0)	
30–39	400 (32.9)	143/257	948 (34.0)	
40–49	295 (24.3)	102/193	545 (19.6)	
50–64	237 (19.5)	85/152	348 (12.5)	
Cohabitation				
Living with partner/husband/wife	777 (64.0)	315/465		
Level of education				47.2 (< 0.01)
0–9 years	489 (40.2)	176/313	1366 (49.1)	
9–12 years	255 (21.0)	86/169	637 (22.9)	
> 12 years	471 (38.8)	190/281	790 (28.4)	
Years since immigration				34.0 (< 0.01)
Five or more	79 (6.5)	22/57	324 (11.6)	
Four	334 (27.5)	117/217	845 (30.4)	
Three	802 (66.0)	3313/489	1615 (58.0)	
Place of residence				
Big cities and surrounding municipalities	337 (27.7)	109/228		
Large towns and surrounding municipalities	573 (47.2)	227/346		
Smaller villages and rural municipalities	305 (25.1)	116/189		
Low social support^a^	653 (53.7)	224/429		

^a^Score of ≤ 3 on two or more items and a total score of ≤ 18 on ESSI (item #4 and #7 excluded)

### Distribution of reported problems in the EQ-5D-5L dimensions

The distribution and severity of problems reported in each of the five dimensions, for the whole sample as well as split by sex and age group, are presented in Table 2. Almost a third of the study population (27.8%) reported *No problem* in all five dimensions of EQ-5D-5L. The anxiety/depression dimension of EQ-5D-5L was the dimension in which most respondents reported problems. Almost two-thirds of the population reported that they were either anxious or depressed (61.9%), where about a tenth of the total respondents (10.2%) reported either severe or extreme anxiety or depression. Within the dimension of pain/discomfort, about half of the study population (54.8%) reported some level of pain/discomfort. In total, the vast majority (93.4%) stated that they had no problems with self-care (washing or dressing one-self).

**Table 2:** Distribution in valid percentages of respondents reporting no problems (Level 1) or problems (Level 2–5) in each of the five EQ-5D-5L dimensions, by sex and age group

EQ-5D dimension	Level of problems^a^	Total	Sex		Age groups			
			Women (%) (*n* = 452)	Men (%) (*n* = 763)	18–29% (*n* = 283)	30–39% (*n* = 400)	40–49% (*n* = 295)	50–64% (*n* = 237)
Mobility	Level 1	72.9	67.6	76.0	88.2	79.1	70.1	47.2
	Level 2	13.1	13.5	12.9	8.2	10.8	14.2	21.6
	Level 3	9.6	12.6	7.8	1.8	6.7	11.8	21.2
	Level 4	4.2	6.3	3.0	1.4	3.4	3.8	9.5
	Level 5	0.2	0.0	0.3	0.4	0.0	0.0	0.4
Self-care	Level 1	93.4	92.1	94.2	97.2	95.3	94.4	84.1
	Level 2	3.5	4.5	2.8	1.8	2.1	3.8	7.5
	Level 3	1.7	1.8	1.6	1.1	1.3	0.7	4.4
	Level 4	1.3	1.6	1.1	0	1.3	1.0	3.1
	Level 5	0.2	0	0.3	0	0	0	0.9
Usual activities	Level 1	70.5	67.2	72.5	81.9	76.2	70.7	47.0
	Level 2	12.6	13.2	12.3	8.2	11.4	14.5	17.7
	Level 3	11.3	13.6	9.8	7.1	7.0	10.3	24.6
	Level 4	4.1	4.5	3.9	1.8	4.4	2.8	8.2
	Level 5	1.5	1.6	1.5	1.1	1.0	1.7	2.6
Pain/discomfort	Level 1	45.2	37.8	49.6	59.4	50.6	39.7	25.5
	Level 2	25.9	28.6	24.3	22.8	24.7	30.7	26.0
	Level 3	19.1	22.6	17.1	11.4	17.2	20.3	30.3
	Level 4	6.7	8.3	5.8	3.6	5.1	6.9	13.0
	Level 5	3.0	2.7	3.2	2.8	2.3	2.4	5.2
Anxiety/depression	Level 1	38.1	36.2	39.3	42.9	40.1	37.8	29.4
	Level 2	30.9	31.0	30.8	30.1	30.2	34.0	29.0
	Level 3	20.7	22.5	19.6	19.1	20.4	17.4	27.3
	Level 4	6.7	6.7	6.8	5.0	7.0	6.9	8.2
	Level 5	3.5	3.6	3.5	2.8	2.3	3.8	6.1

^a^Level 1 = no problems, Level 2 = slight problems, Level 3 = moderate problems, Level 4 = severe problems, Level 5 = extreme problems

Differences between women and men were found in the EQ-5D-5L dimensions of pain/discomfort and mobility (Table 3): women had an increased risk of experiencing problems with mobility (OR 1.524, 95% CI 1.174‒1.977) and pain/discomfort (OR 1.619, 95% CI 1.274‒2.056) compared to men.

Regarding differences between age groups, the risk of problems in all five dimensions increased with age (See Table 3): the oldest age group had a four- to eightfold risk of experiencing problems with mobility, self-care, usual activities and pain/discomfort compared to the youngest age group. The risk of experiencing problems in the dimension anxiety/depression was also elevated for the oldest age group compared to the youngest (OR 1.802, 95% CI 1.246‒2.604). In the oldest age group (50‒64 years), almost 75% reported some level of pain/discomfort, where almost one-fifth (18.2%) reported severe or extreme pain/discomfort. It was also shown that about 70% of the respondents in this age group reported that they were slightly to extremely anxious/depressed.

**Table 3:** Risk of having problems in each EQ-5D dimension by sex and age in odds ratios (OR) with 95% confidence intervals (CI)

EQ-5D dimensions															
	Mobility			Self-care			Usual activities			Pain/discomfort			Anxiety/depression		
	OR	95% CI		OR	95% CI		OR	95% CI		OR	95% CI		OR	95% CI	
Sex															
Men (ref)	1			1			1			1			1		
Women	1.524	1.174	1.977	1.391	0.876	2.21	1.288	0.998	1.662	1.619	1.274	2.056	1.144	0.898	1.458
Age groups															
18‒29 (ref)	1			1			1			1			1		
30‒39	1.975	1.274	3.06	1.684	0.722	3.931	1.417	0.966	2.079	1.428	1.047	1.947	1.125	0.824	1.536
40‒49	3.187	2.048	4.959	2.03	0.854	4.821	1.878	1.265	2.787	2.229	1.595	3.116	1.234	0.883	1.726
50‒64	8.378	5.365	13.083	6.489	2.951	14.272	5.111	3.433	7.61	4.271	2.921	6.243	1.802	1.246	2.604

### HRQoL index values

There were 111 women (25%) and 212 men (28%) who had an index value of 1, which indicates the best possible health status. The mean EQ-5D-5L index value for the total population was 0.754. Women had a mean index value of 0.735, and men a mean value of 0.765 (Table 4). Regarding age, the highest index value was found in the youngest age group (18‒29 years), with a value of 0.813, and lowest (0.635) in the oldest age group (50‒64 years). The highest mean index value was found among women in the youngest age group (0.845), and the lowest mean index value was found among women in the oldest age group (0.542). Corresponding values for men were 0.790 in the youngest age group and 0.689 in the oldest age group.

**Table 4:** Mean, standard error of mean, median, 25th and 75th quantile for computed EQ-5D-5L index values, using the UK crosswalk tariff, for the study population and by sex and age category

EQ index	Total	Age groups			
		18‒29	30‒39	40‒49	50‒64
Total					
Mean	0.754	0.813	0.782	0.750	0.635
Std error	0.007	0.014	0.012	0.015	0.019
Median	0.768	0.879	0.837	0.768	0.696
25th	0.681	0.736	0.722	0.689	0.486
75th	1	1	1	1	0.837
*N*	1163	277	380	283	223
Women					
Mean	0.735	0.845	0.783	0.699	0.542
Std error	0.012	0.015	0.019	0.028	0.032
Median	0.768	0.879	0.795	0.767	0.620
25th	0.664	0.747	0.702	0.653	0.367
75th	1	1	1	0.848	0.743
*N*	436	118	137	99	82
Men					
Mean	0.765	0.790	0.782	0.778	0.689
Std error	0.009	0.021	0.016	0.016	0.023
Median	0.819	0.879	0.848	0.773	0.760
25th	0.708	0.725	0.725	0.709	0.609
75th	1	1	1	1	0.879
*N*	727	159	243	184	141

### Factors associated with HRQoL index value

In our first model which included the variables sex and age group, associations were found between EQ-5D-5L index value and both sex and age (Table 5). To be a woman was associated with a lower index value in comparison with being a man. Being older was associated with a lower index value compared to being younger.

**Table 5:** Multiple linear regression analysis of associations between EQ-5D-5L index values and included variables, ordinary least squares regression and unstandardized and (standardized) regression coefficient

Covariates	Model 1^a^			Model 2^b^			Model 3^c^		
	Unstandardized β	Standardized β	*P* value	Unstandardized β	Standardized β	*P* value	Unstandardized β	Standardized β	*P* value
Sex									
Woman	− 0.033	− 0.062	0.029	− 0.045	− 0.085	0.003	− 0.048	− 0.091	0.001
Man	Ref.			Ref.			Ref.		
Age group									
18‒29	0.180	0.301	< 0.001	0.221	0.369	< 0.001	0.200	0.334	< 0.001
30‒39	0.147	0.270	< 0.001	0.154	0.283	< 0.001	0.143	0.263	< 0.001
40‒49	0.115	0.194	< 0.001	0.111	0.186	< 0.001	0.101	0.169	< 0.001
50‒64	Ref.			Ref.			Ref.		
Educational level									
0‒9				Ref.					
10‒12				0.017	0.028	0.374	0.024	0.037	0.220
> 12				0.023	0.045	0.155	0.020	0.039	0.211
Cohabitation									
Living with partner/marital partner				0.084	0.157	< 0.001	0.066	0.124	< 0.001
Not living with partner/marital partner				Ref.					
Place of residence									
Large cities and surrounding municipalities				Ref.					
Large towns and surrounding municipalities				0.030	0.058	0.086	0.023	0.044	0.185
Smaller villages and rural municipalities				0.029	0.049	0.149	0.026	0.045	0.184
Social support									
Low social support							− 0.094	− 0.184	< 0.001
Social support							Ref.		
R^2^	0.063			0.089			0.121		

^a^Model 1 includes variables sex and age

^b^Model 2 includes variables sex, age, educational level, cohabitation and place of residence

^c^Model 3 includes variables sex, age, educational level, cohabitation, place of residence and social support

In the second model, which included sex, age, educational level, cohabitation and place of residence, associations were found between EQ-5D-5L index value and sex, age and cohabitation. Educational level and place of residence were not associated with the EQ-5D-5L index value.

In the third model, all the above-mentioned variables were included with the addition of the variable social support. The associations above persisted, and social support was found to be positively associated with the EQ-5D-5L index value, i.e., experiencing low social support was associated with a lower EQ-5D-5L index value. The final model accounted for 12.1% of the total variance.

## Discussion

There is currently a lack of knowledge regarding the health status among refugees resettled in high-income countries. The main aim of this study was, thus, to assess the level of HRQoL among Syrian refugees resettled in Sweden. In the same line, the distribution of problems in the five different dimensions of the EQ-5D in this population was examined. We also wanted to investigate possible differences in HRQoL between women and men and across age groups, and explore whether educational level, cohabitation, place of residence after resettlement, and social support were associated with HRQoL. This is the first study, to our knowledge, that investigates HRQoL among Syrian refugees in a high-income country, using a highly standardized measure of HRQoL, EQ-5D-5L, which provides the possibility to compare the health status of this refugee population to other refugee and non-refugee populations.

Depression/anxiety and pain/discomfort were the two particular dimensions in which most health problems were reported in this study population. A majority of the study population reported some level of problems in these two dimensions, and about 10% reported either severe or extreme problems. Women had an increased risk of experiencing problems with mobility and pain/discomfort compared to men. These results suggest that predominant health problems among the study population appear to be those which are generally related to exposure to traumatic experiences and distressful life conditions, i.e., mental health [4] and pain-related distress [23]. As it has been shown in previous research, these health sequelae of refugee-related exposures seem to be persistent long after resettlement [4].

Our results show that the mean index value of the whole population was 0.754. On average, men and younger individuals had a higher level of HRQoL than women and older individuals. Interestingly, the results of the stratified analyses on both sex and age revealed that the highest mean HRQoL index value in the study population was found among the women in the youngest age group. This indicates that the overall low HRQoL among women in the study population could be ascribed to poor health status among women in the older age groups, and possibly suggests that there may be a steeper age-related decline in HRQoL among women compared to men. This, however, needs to be more closely investigated by means of longitudinal studies. Moreover, the results of the regression models corroborated the patterns of differences between men and women and across age groups, beyond which also cohabitation and social support emerged as correlates of HRQoL, indicating these factors embedded in the living conditions of resettled refugees assert an impact on the health status of refugees.

Our study population consisted of Syrian refugee women and men who had been resettled in Sweden since 3 to 5 years. In comparison with the most recent data from the Swedish general population in 2002, the mean HRQoL index value was lower for both women and men in our study population (0.797 vs. 0.735 for women and 0.841 vs. 0.765 for men, respectively) [18]. The two EQ-5D dimensions pain/discomfort and anxiety/depression were also the two dimensions accounting for the most frequently reported problems among the Swedish general population, which indicates that pain/discomfort and anxiety/depression may act as key drivers of low HRQoL across different non-patient populations. In previous studies from Sweden and Finland, comparing HRQoL between elderly Iranian migrants/Somali refugees and the Swedish/Finnish host population, similar results were found [15, 16]. Sex differences in HRQoL were also shown in a study with data from Sweden, where female Iranian migrants had lower scores in all dimensions compared to Swedish women, whereas male Iranian migrants had lower score in six of the eight dimensions measured by SF-36 [16]. These findings are, furthermore, in line with previous research comparing health between immigrants born outside EU, not specifically refugees, and the Swedish host population, where differences in reported poor health were multifold [24]. Taken together, our results indicate that worse health among refugee populations compared to host society’s general population may not merely be limited to mental health but seem to extend to health status in general and HRQoL.

Furthermore, this study provides some evidence of the importance of social support for overall health among refugees. This is in line with previous research among general population [12, 13], immigrants in Sweden [25] and refugee torture-survivors in Denmark [26], as well as a recently published study among Syrian refugees from our research group [22]. The stress buffering model [16] which suggests that supportive social networks enhance individuals’ coping with stressful life events to buffer against the development of stress-related psychopathology, in light of our results, may be extended to be applicable to HRQoL among refugee populations that face multiple pre- and post-migratory stressful living conditions.

Sex and age are well-known determinants of health, where women tend to have worse health but live longer than men, and older tend to have worse health than younger [27–29]. Similar associations have also been shown regarding HRQoL [30]. Worth highlighting is, however, the extremely elevated risk of poor health found among the oldest age group in most of the measured dimensions of health of this study. Another important and somewhat surprising finding in this study is the lack of importance of educational level for HRQoL. Education is one of the measures used for socioeconomic position, which often is found associated with different outcomes in health [31]. As education is expected to contain an individual’s potential for both income and employment, it has been assessed as a good marker for an individual’s living conditions, which in turn would influence health [31]. In contrast, a study by Porter and Haslam [9] found pre-migration high educational level associated with mental ill health, which could be explained by the higher likelihood of loss of status in the host country implied in pre-arrival higher social position. In regard to HRQoL, our results neither show patterns confirming to a positive or negative association between education level and health status. This could, perhaps, be viewed as either suggesting that high education does not function as a marker for socioeconomic position among newly resettled refugees or that other refugee-related living conditions such as pre-migratory trauma, and post-resettlement social support override the potential effect of educational level on health.

Whereas sex, age, and cohabitation are demographic and inherent subject factors, our study shows social support, a modifiable factor, to also be relevant to refugees’ overall health. Facilitating, mobilizing and enhancing refugees’ social support may therefore be considered a target of public health-level interventions. In contrary, it is worth pointing out that policies and regulations that constrain and impede access to close supportive relationships, such as policies restricting family reunification, may risk impacting negatively on refugees’ resources for HRQoL.

Our overall results concerning long-lasting health problems among the study population indicate that there is a profound need for policies and interventions promoting refugees’ health, e.g., allocation of resources to specialist care for rehabilitation of trauma-afflicted refugees. Given the large scale of the health challenges faced by refugees as indicated by our results and previous research [2], however, there is also a need for public health-level and scalable health-promoting policies that prevent the health of refugees from deteriorating in post-resettlement. This is particularly important given the complexity and persistence of health problems among those refugees who have been through severe or multiple traumatic events [32].

### Strengths and limitations

A unique strength of this study, one of the larger studies of HRQoL within this research area, is self-reported data from a large random sample of Syrian refugees selected from a complete and known sample frame. The study population consists of refugees who are generally considered hard to reach populations [33], and this is reflected in the response rate of 30.4%. Although within-subject associations analyses have been suggested to be less prone to non-response bias [34, 35], a low response rate might imply a risk of selection bias leading to, i.e., an overestimation of HRQoL [36]. Due to this important limitation, which is inherent in research with hard to reach populations, the risk of bias in estimating finite population characteristics should be acknowledged. Although our previous studies have shown that the socio-demographic characteristics of the sample corresponds closely to that of the randomly selected sample frame from the target population, the results should be viewed cautiously in regard to its generalizability to other refugee populations.

Furthermore, given that the study has a cross-sectional design, causal directions in association analyses should not be assumed, although the socio-demographic variables could be viewed as antecedents in this regard as they remain non-modifiable.

Another strength of our study is the use of validated, standardized instrument for assessment of HRQoL and also for assessment of social support. However, previous comparisons of HRQoL between countries have suggested that the value might differ depending on the norm-data used [37]. Furthermore, information about the socio-demographic variables, except civil status, was retrieved from national, high-quality registers, reducing the risk of information bias. Finally, the assessment lacks data on EQ-5D VAS due to technical difficulties. Although this constitutes a limitation, on basis of previous research on correspondence between index and VAS values [38] it is possible that the available data approximate the evaluation that VAS could have provided.
